# Fixed-Dose Combination Drug Approvals, Patents and Market Exclusivities Compared to Single Active Ingredient Pharmaceuticals

**DOI:** 10.1371/journal.pone.0140708

**Published:** 2015-10-15

**Authors:** Jing Hao, Rosa Rodriguez-Monguio, Enrique Seoane-Vazquez

**Affiliations:** 1 School of Public Health and Health Sciences, University of Massachusetts, Amherst, Massachusetts, United States of America, 01003; 2 Massachusetts College of Pharmacy and Health Sciences, Boston, Massachusetts, United States of America, 02115; 3 Brigham and Women’s Hospital, Division of General Medicine and Primary Care, Boston, Massachusetts, United States of America; UNAIDS, TRINIDAD AND TOBAGO

## Abstract

**Introduction:**

Fixed-dose combinations (FDC) contain two or more active ingredients. The effective patent and exclusivity life of FDC compared to single active ingredient has not been assessed.

**Objectives:**

Trends in FDA approved FDC in the period 1980–2012 and time lag between approval of FDC and single active ingredients in the combination were assessed, and the effective patent and exclusivity life of FDC was compared with their single active ingredients.

**Materials and Methods:**

New molecular entities (NMEs), new therapeutic biologics license applications (BLAs) and FDC data were collected from the FDA Orange Book and Drugs@FDA. Analysis included FDC containing one or more NMEs or BLAs at first FDA approval (NMEs-FDC) and only already marketed drugs (Non-NMEs-FDC). Descriptive, Kruskal-Wallis and Wilcoxon Rank Sum analyses were performed.

**Results:**

During the study period, the FDA approved 28 NMEs-FDC (3.5% of NMEs) and 117 non-NMEs-FDC. FDC approvals increased from 12 in the 1980s to 59 in the 2000s. Non-NMEs-FDC entered the market at a median of 5.43 years (interquartile range 1.74, 10.31) after first FDA approval of single active ingredients in the combination. The Non-NMEs-FDC entered the market at a median of 2.33 years (-7.55, 2.39) before approval of generic single active ingredient. Non-NME-FDC added a median of 9.70 (2.75, 16.24) years to the patent and exclusivity life of the single active ingredients in the combination.

**Conclusion:**

FDC approvals significantly increased over the last twenty years. Pharmaceutical companies market FDC drugs shortly before the generic versions of the single ingredients enter the market extending the patent and exclusivity life of drugs included in the combination.

## Introduction

Patents are one of the forms of intellectual property regulation for new drugs [1–5]. The Uruguay Round of the General Agreement on Tariffs and Trade (GATT), negotiated in 1994, includes the Agreement on Trade-Related Aspects of Intellectual Property Rights (TRIPS) that established minimum intellectual property regulation standards to World Trade Organization (WTO) members [6]. In the US, the Uruguay Round Agreements Act (URAA), enacted in June 8, 1995, approved and implemented the TRIPS. URAA established a 20-year patent term (i.e. patent statutory term) from the filling date of a patent application before the United States Patent and Trademark Office (USPTO). Before URAA, patentees had 17 years of patent life upon the date when the patent was issued by the USPTO.

Patent extensions are granted by the USPTO to partly restore the time spent on clinical trials and FDA review and market exclusivity are recognized by the FDA upon approval of certain drug applications [7–9]. Pharmaceutical products do not face generic competition during the effective patent and exclusivity life period thus, pharmaceutical companies set up prices of new drugs to maximize profits [5,10]. Once the patents and exclusivities expire, generic drugs may enter the market driving down pharmaceutical prices.

Fixed-dose combination drugs (FDCs) are formulations that contain two or more active ingredients in a single dose [11]. According to the FDA, “two or more drugs may be combined in a single dose when each component makes a contribution to the claimed effects, and the dosage of each component (i.e., amount, frequency, and duration) is such that the combination is safe and effective for a significant patient population requiring such concurrent therapy” [12].

**Fig 1:**
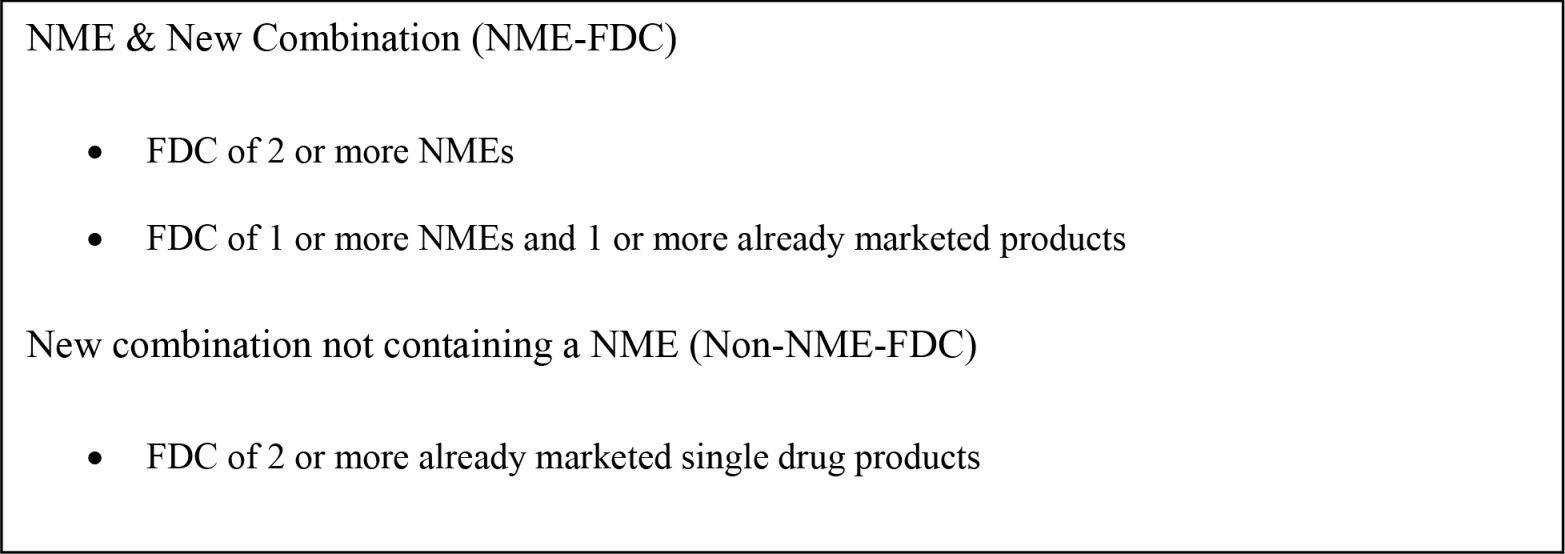
Classification of FDC at First Approval

A new molecular entity (NME) and a new biologic license application (BLA) are drugs containing active substances that have never before been approved for marketing in the US. Some new drugs are first introduced as a FDC in the US market (i.e. NME-FDC). A NME-FDC may contain only NMEs or a mix of NMEs and other already marketed drugs. A Non-NME-FDC is a new combination that contains only already marketed drugs (Fig 1). The development and marketing of FDCs have been a strategy of brand-name drug companies to extend the drug patent and exclusivity life of pharmaceuticals in the US, particularly after the enactment of the Waxman-Hatch Act (WHA) in 1984 [13, 14].

A patent confers the right to exclude others from making, using, offering for sale, selling or importing an invention. The Drug Price Competition and Patent Restoration Act of 1984 (Waxman-Hatch Act), and the Food and Drug Administration Modernization Act of 1997 (FDAMA) include provisions that increase the patent and market exclusivity periods for pharmaceuticals.

The Waxman-Hatch Act provides patent term extensions for patents covering new drugs, allowing sponsor companies to recover the time spent during the FDA New Drug Application (NDA) review and half of the time spent in clinical trials and administrative activities required for FDA approval. The patent term extension can be applied to a single patent with a limit of five years; once the extension is added. The patent time remaining from NDA approval cannot exceed fourteen years. The Waxman-Hatch Act also includes 5 years exclusivity for NMEs and 3 years exclusivity to certain NDAs that contain active ingredients previously approved by the FDA for marketing in the US. The Food and Drug Administration Modernization Act provides a six-month exclusivity period for drug manufactures that conduct FDA-approved pediatric research. The six-month exclusivity applies to all formulations, dosage forms, and indications for products containing the active moiety for which the submitting company holds an approved NDA, and it is added to all non-expired patents and exclusivity periods.

The effective patent and exclusivity life of a drug is the period of time from FDA NDA approval to patent expiration. Throughout the duration of the effective patent life, the NDA sponsor company has the right to exclude competitors from marketing the drug in the US. Additionally, the FDA does not approve generic drug competitors during the period of exclusivity of a NDA.

**Fig 2:**
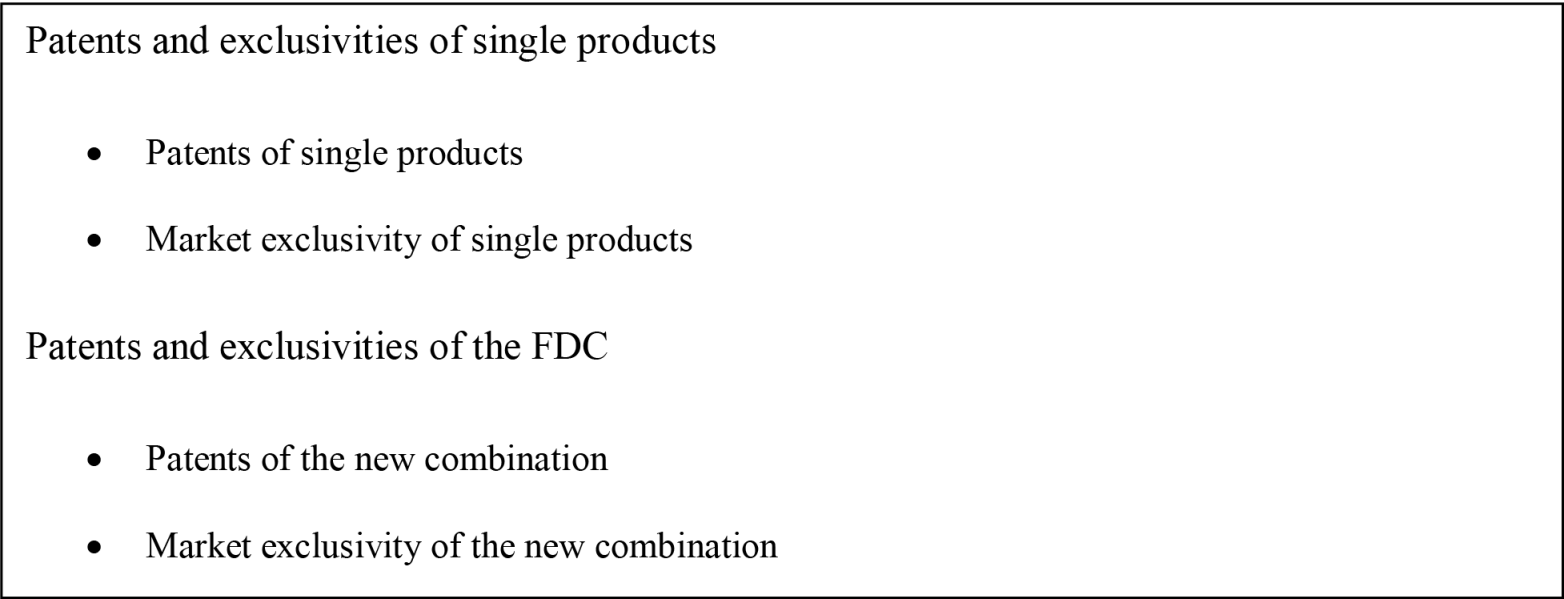
FDC Potential Patent and Exclusivity Protection

In the US, if a FDC is novel, non-obvious, and useful, it can be patented and the exclusion of competitors from the market can be enforced (Fig 2). In this case, the sponsor company is able to add patent and exclusivity time to the combination of individual products included in the FDC, for which patents and exclusivities may be expired or close to expire. The FDA provides three years of market exclusivity to new NME-FDC when the application contains new clinical investigations. If the new FDC is not patentable, the patent and exclusivity life of the FDC will typically be equal to the three year market exclusivity or the longest patent and exclusivity life of its individual components. Patents claiming a FDC are classified as *secondary patents*. A secondary patent claims features, other than the original active drug ingredient, including combinations and other formulations and methods of administration [15,16]. In this case, the sponsor company is able to add patent time to the combination of individual products included in the FDC, for which patents may be expired or close to expire. A patent for a FDC applies only to the combination and does not extend the patent life of the individual active ingredients of the FDC.

FDC drugs allow patent holders to maintain the market share for products included in the combination, to expand their patent and exclusivity protection, and to shift the demand from single active ingredients to the FDC as patent expiration of single active ingredients looms [14,17]. The substitution of less-expensive single drug products with newer, high-priced, combinations leads to increases in pharmaceutical expenditures [18].

To the best of authors’ knowledge, no empirical analysis has been conducted to assess the extent to which FDC drugs expand the effective patent and exclusivity life of pharmaceuticals. Due to the growing number of FDC approved by the FDA and the difference in cost between FDC and single active ingredients, there is a need for an in-depth analysis of trends in FDC drugs approvals and the effective patent and exclusivity life of FDC compared to single active ingredients included in the combination. Thus, the objectives of this study were: 1) to assess trends in FDCs and single active ingredients approved by the FDA in the period 1980–2102; 2) to estimate the time lag between the first approval of single active ingredients and the FDC drugs containing those active ingredients; 3) to estimate the time lag between the first FDC approval and the approval of abbreviated new drug applications (ANDAs) for the active ingredients included in the combination; and 4) to estimate the effective patent and exclusivity life of FDC drugs compared to the single active ingredients included in the combination.

## Materials and Methods

Data were derived from the electronic versions of the FDA Approved Drug Products with Therapeutic Equivalence Evaluations (FDA Orange Book) [19] and the Drug@FDA database [20] from 1980 to 2012. The study included all FDA approved NMEs and BLAs during the study period.

Information collected for each FDA-approved pharmaceutical product included the NDA number, product number, generic name, trade name, dosage form/route of administration, Anatomical Therapeutic Chemical (ATC) code, National Drug Code (NDCs), market status (i.e., prescription, over-the-counter or discontinued), NDA approval date, patent expiration date, and market exclusivity data. Therapeutic category information was extracted from the ATC classification system maintained by the World Health Organization Collaborating Centre for Drug Statistics Methodology [21].

Using FDA data, a dataset with all NDAs and BLAs approved by the FDA during the study period was created. All NMEs and BLAs approved during the study period, and all FDCs containing at least one of those NMEs and BLAs were selected. The analysis was broken down into two groups; FDCs containing at least one NME/BLA at first FDA approval (NME-FDC) and FDCs containing single active ingredients approved for the first time during the study period (non-NME-FDC). The units of analysis were the first NDA/BLA of all NMEs, BLAs, NME-FDCs, and non-NME-FDCs approved for the first time by the FDA during the study period.

The effective patent and exclusivity life is the time period from the FDA approval of the NDA to the expiration of all pharmaceutical patents and market exclusivities [22]. The time lag from the last approval of the single active ingredients and the first approval of FDCs containing those active ingredients was calculated. The time lag from the first ANDA approval of the single active ingredients and the first approval of FDCs containing those active ingredients was also calculated.

When a FDC had a generic alternative, the time difference in the patent and exclusivity life of the FDC and the single active ingredients included in the combination was estimated as the time between the dates of FDC first ANDA approval and the single active ingredients ANDA approvals. When a FDC did not have a generic alternative in the market, the difference in the patent and exclusivity life was estimated as the time between the FDC last patent and market exclusivity expiration date and the single active ingredients ANDA approval dates. The analysis was stratified by pharmaceutical sponsors that marketed both the single active ingredients and the corresponding FDC drugs, and by sponsors that marketed FDC drugs but not the single active ingredients.

Descriptive analyses including median and interquartile range (IQR) were performed for study variables including approvals of FDCs and single ingredient drugs, the time lag between the FDC approvals and the single ingredient drugs and the effective patent and exclusivity life of the FDCs compared to the single ingredient drugs. Differences among therapeutic categories were tested using non-parametric analysis of variance Kruskal-Wallis test; the two groups Wilcoxon Rank Sum tests were also used to identify the significant differences between pairs of two specific therapeutic classes. Bonferroni Correction was applied to adjust for multiple testing. Differences between non-NME-FDC and the single active ingredients sponsored by the same company and those sponsored by different companies were tested by two-group Wilcoxon Rank Sum tests. Inferential analyses, which employ probability theory and test significances, were performed on therapeutic classes that had 5 or more FDC drugs. Significance level was set at 0.05. All analyses were performed using SAS software, version 9.4 (SAS Institute, Inc., Cary, NC, USA).

## Results

### FDA-approved FDC Drugs

In the period 1980–2012, the FDA approved 901 new drugs, including 811 NMEs and 90 BLAs. NME-FDC drugs (n = 28) represented 3.5% of the FDA-approved NMEs (n = 811). The FDA did not approve any combination for BLAs. In the study period, 7 (25%) of the 28 NME-FDC drugs were discontinued. The largest number of NME-FDC drugs were antiinfectives (n = 7), genito-urinary system and sex hormones (n = 6), and dermatologicals (n = 4). The majority (5 out of 7) of the FDC antiinfectives was approved in the 1980s and 1990s (Table 1).

**Table 1:** FDA-Approved NMEs and BLAs and NNE-FDC drugs by Therapeutic Category, 1980–2012.

APPROVALS	1980s	1990s	2000s	2010–2012	Total
NME-FDC Approvals by Therapeutic Class					
Alimentary tract and metabolism	1	0	1	1	3
Antiinfectives for systemic use	3	2	1	1	7
Antineoplastic and immunomodulating	0	0	1	0	1
Antiparasitic products, insecticides and repellents	1	0	0	0	1
Cardiovascular system	0	0	0	0	0
Dermatologicals	1	1	2	0	4
Genito urinary system and sex hormones	1	1	3	1	6
Musculo-skeletal system	0	0	0	0	0
Nervous system	0	0	1	0	1
Respiratory system	0	2	0	0	2
Systemic hormonal preparations	0	0	1	0	1
Various	1	0	1	0	2
NME-FDC Approvals	8	6	11	3	28
Single Active Ingredient BLA and NME Approvals	217	333	240	83	873
Total NME-FDC and Single Active Ingredient BLA and NME Approvals	225	339	251	86	901
**AVERAGE APPROVALS PER YEAR**	** **	** **	** **	** **	** **
NME-FDC	0.8	0.6	1.1	1.0	0.8
Single Active Ingredient BLA and NME	21.7	33.3	24.0	27.7	26.5
NME-FDC and Single Active Ingredient BLA and NME	22.5	33.9	25.1	28.7	27.3

In addition, the FDA approved 117 non-NME-FDC drugs (i.e. 115 NMEs and 2 BLAs) that had at least one single active ingredient approved by the FDA during the study period (Table 2). The non-NME-FDC drugs approved in the study period included 156 different single active ingredients with an average of 2.1 active ingredients per combination. A total of 23 (20%) of the 117 non-NME-FDC drugs were discontinued from the market as of December 31, 2012. Non-NME-FDC drugs approved by the FDA increased over time from an average of 1.2 approvals per year (n = 12) in the 1980s to 2.5 (n = 25) in the 1990s and 5.9 (n = 59) in the 2000s. During the period 2010–2012, the FDA approved an average of 7.0 (n = 21) non-NME-FDC drugs per year. The percentage of non-NME-FDC and NME/BLA increased from 5.5% (12/217) in 1980s to 25.3% (21/83) during the period 2010–2012. The ATC classes with the largest number of non-NME-FDC approved by the FDA were cardiovascular diseases (n = 41), alimentary tract and metabolism (n = 26), respiratory system (n = 10), and antiinfectives (n = 10).

**Table 2:** FDA-Approved Non-NNE-FDC drugs by Therapeutic Category, 1980–2012.

APPROVALS	1980s	1990s	2000s	2010–2012	Total
Non-NME-FDC Approvals by Therapeutic Class					
Alimentary tract and metabolism	2	2	14	8	26
Antiinfectives for systemic use	2	2	5	1	10
Antineoplastic and immunomodulating	0	1	2	1	4
Antiparasitic products, insecticides and repellents	0	0	1	0	1
Cardiovascular system	7	13	15	6	41
Dermatologicals	0	1	4	0	5
Genito urinary system and sex hormones	0	0	1	2	3
Musculo-skeletal system	0	1	2	0	3
Nervous system	0	0	5	0	5
Respiratory system	1	2	5	2	10
Systemic hormonal preparations	0	3	3	1	7
Various	0	0	2	0	2
Non-NME-FDC Approvals	12	25	59	21	117
**AVERAGE APPROVALS PER YEAR**	** **	** **	** **	** **	** **
Non-NME-FDC Approvals	1.2	2.5	5.9	7.0	3.5

Overall, 10.4% (n = 12) of the 117 non-NME-FDC, were approved by the FDA using the priority review procedure (i.e., a review process applied by the FDA to drugs considered improvements over already marketed therapeutic alternatives). The percentage of priority review approvals was highest for non-NME-FDC of antiinfectives for systemic use (n = 5) (50.0% of total 10 FDA approvals).

### Market Entry and Effective Patent Life

Non-NME-FDC entered the market at a median of 5.43 years (interquartile range—IQR 1.74, 10.31 years) after the first approval of the single active ingredients included in the combination (Table 3). This time lag significantly varied by therapeutic class (Kruskal Wallis test; p = 0.015). Antiinfectives and cardiovascular system non-NME-FDC entered the market significantly sooner (median 1.89 years, IQR 0.22, 5.63 years) compared to the nervous system (7.23, IQR 6.45, 20.77), respiratory system (9.34, IQR 5.67, 9.82) and sensory organs (10.73, IQR 6.77, 11.63) (Wilcoxon Rank Sum test; p<0.05) (Table 3). The difference in the time lag between approval of Non-NME-FDC and the first approval of the single active ingredient included in the combination by therapeutic class was not statistically significant after applying Bonferroni Correction.

**Table 3:** Time Lag between Approval of Single Active Ingredient NME and FDC.

Therapeutic Class	FDC and Previously Approved Single Drug NME Same Applicant		FDC and Previously Approved Single Drug NME Different Applicant		Total	
	No. of FDC	Median (IQR)	No. of FDC	Median (IQR)	No. ofFDC	Median (IQR)
Alimentary Tract And Metabolism	15	6.12 (2.57, 7.38)	7	15.56 (1.26, 19.27)	22	6.31 (1.33, 9.51)
Antiinfectives for Systemic Use	9	1.91 (1.09, 5.63)	1	0.22	10	1.89 (0.22, 5.63)^§^
Antineoplastic and Immunomodulating Agents	4	5.59 (2.71,17.54)	0		4	5.59 (2.71,17.54)
Antiparasitic Products, Insecticides & Repellents	1	7.63	0		1	7.63
Cardiovascular System	33	2.68 (1.11, 5.42)	8	7.47 (3.06, 12.41)	41	3.48 (1.14, 7.17)^§^
Dermatologicals	1	12.52	1	27.34	2	19.93 (12.52,27.34)
Genito Urinary System and Sex Hormones	1	8.56	0		1	8.56
Musculo-Skeletal System	1	9.52	2	12.36 (9.41,15.30)	3	9.52 (9.41,15.30)
Nervous System	4	6.84 (5.05,14.00)	1	24.73	5	7.23 (6.45, 20.77)^§^
Respiratory System	6	6.11 (4.11, 9.82)	3	9.63 (9.34,12.43)	9	9.34 (5.67, 9.82)^§^
Sensory Organs	4	11.39 (8.96, 13.34)	2	6.82 (3.33,10.31)	6	10.73 (6.77,11.63)^§^
Various	1	0.00	0		1	0.00
**Total**	80	4.50 (1.69, 7.88)*	25	10.31 (3.52,15.56)*	105	5.43 (1.74,10.31)

Notes: IQR = Interquartile Range.

§Statistically significant difference among therapeutic classes: Antiinfectives for systemic use compared to nervous system (p = 0.028), respiratory system (p = 0.041), and sensory organs (p = 0.030), respectively. Cardiovascular system compared to nervous system (p = 0.027), respiratory system (p = 0.021), and sensory organs (p = 0.018), respectively.

* Statistically significant difference between FDC and previously approved single drug NME same and different applicant (p = 0.011).

The difference in market entry between non-NME-FDC and the single active ingredients sponsored by the same company and those sponsored by different companies was statistically significant. When the non-NME-FDC and the single active ingredient were sponsored by the same company, the FDC entered the market at a median of 4.50 years (IQR 1.69, 7.88) after the first approval of the single active ingredients included in combination; whereas, when the applicant of the non-NME-FDC and the single active ingredient were different, the non-NME-FDC entered the market at a median of 10.31 years (IQR 3.52, 15.56) after the first approval of the single active ingredients in the combination (Wilcoxon Rank Sum test; p = 0.011) (Table 3).

Non-NME-FDC drugs entered the US market at a median of 2.33 years (IQR -7.55, 2.39 years) before the generic alternative of the single active ingredient included in the combination reached the market; the time difference did not significantly varied by therapeutic class (Kruskal Wallis test; p = 0.097) (Table 4). When a non-NME-FDC and the single active ingredients were sponsored by the same company, the non-NME-FDC entered the market at a median of 5.05 years (IQR -8.99, -1.48) before the first generic approval of the active ingredients; whereas, when the sponsor of the non-NME-FDC and the single active ingredients were different, the non-NME-FDC entered the market 1.85 years (IQR -2.12, 5.04) after the generic single active ingredients reached the market (Wilcoxon Rank Sum test; p<0.0001).

**Table 4:** Time lag between FDC Drug Approval and Single Drug Generic Market Entry.

Therapeutic Class	FDC and Previously Approved Single Drug NME Same Applicant		FDC and Previously Approved Single Drug NME Different Applicant		Total	
	No. of FDC	Median (IQR)	No. of FDC	Median (IQR)	No. of FDC	Median (IQR)
Alimentary Tract And Metabolism	11	-5.99 (-7.17, -1.48)	9	5.18 (3.31, 8.78)	20	-0.20 (-6.03, 4.29)
Antiinfectives for Systemic Use	6	-6.07 (-7.98, -3.09)	0		6	-6.07 (-7.98, -3.09)
Antineoplastic and Immunomodulating Agents	2	2.91 (-8.54, 14.36)	2	2.18 (0.16, 4.19)	4	2.18 (-4.19, 9.28)
Antiparasitic Products, Insecticides And Repellents	1	-10.50	0		1	-10.50
Cardiovascular System	18	-8.42 (-12.66, -4.97)	17	1.42 (-5.76, 3.57)	35	-5.12 (-10.14, 1.65)
Dermatologicals	1	-1.48	1	12.27	2	5.40 (-1.48, 12.27)
Genito Urinary System And Sex Hormones	1	-0.52	0		1	-0.52
Musculo-Skeletal System	2	-1.82 (-2.83, -0.81)	1	2.49	3	-0.81 (-2.83, 2.49)
Nervous System	4	0.78 (-4.97, 4.37)	1	11.27	5	2.39 (-0.84, 6.35)
Respiratory System	7	-3.72 (-6.38, 0.03)	2	-1.24 (-2.33, -0.15)	9	-2.33 (-4.05, -0.15)
Sensory Organs	2	0.96 (-2.51, 4.42)	3	-6.33 (-10.56, 9.89)	5	-2.51 (-6.33, 4.42)
Various	0		0		0	
Total	55	-5.05 (-8.99, -1.48)***	36	1.85 (-2.12, 5.04)***	91	-2.33 (-7.55, 2.39)

Notes:

*** Statistically significant difference between FDC and single drug NME same and different applicant, p<0.0001. IQR = Interquartile Range

Non-NME-FDC drugs added a median of 9.70 years (IQR 2.75, 16.24) of patent and market exclusivity protection to the effective patent and exclusivity life of the single active ingredients included in the combination; being the difference by therapeutic class not statistically significant (Kruskal Wallis test; p = 0.154) (Table 5). The difference in the effective patent and exclusivity life between non-NME-FDC and the single active ingredients sponsored by the same company and those sponsored by different companies was statistically significant. When the sponsor of the non-NME-FDC was the same as the single ingredient drug, the non-NME-FDC added a median of 7.73 years (IQR 1.07, 11.36) to the patent and market exclusivity life of the single active ingredient. Furthermore, when the sponsor of the non-NME-FDC and the single ingredient drug were different, the non-NME-FDC added a median of 11.48 years (IQR 7.23, 20.71) of patent and market exclusivity protection (Wilcoxon Rank Sum test; p = 0.005).

**Table 5:** Effective Patent and Exclusivity Life: FDCs Compared to Single Active Ingredient Included in Combination.

Therapeutic Class	FDC and Previously Approved Single Drug NME Same Applicant		FDC and Previously Approved Single Drug NME Different Applicant		Total	
	No. of FDC	Median (IQR)	No. of FDC	Median (IQR)	No. of FDC	Median (IQR)
Alimentary Tract And Metabolism	9	9.70 (7.73, 11.36) ^§§^	9	21.51 (11.22, 25.25)	18	11.29 (7.73, 21.51)
Antiinfectives for Systemic Use	4	6.04 (3.93, 7.60)	0		4	6.04 (3.93, 7.60)
Antineoplastic and Immunomodulating Agents	0		0		0	
Antiparasitic Products, Insecticides And Repellents	1	0.00	0		1	0.00
Cardiovascular System	10	1.05 (0.00, 2.74) ^§§^	15	10.84 (5.40, 12.80)	25	6.98 (1.07, 11.06)
Dermatologicals	1	17.13	1	23.85	2	20.49 (17.13, 23.85)
Genito Urinary System And Sex Hormones	1	5.72	0		1	5.72
Musculo-Skeletal System	2	13.80 (10.95, 16.65)	1	17.03	3	16.65 (10.95, 17.03)
Nervous System	4	6.86 (1.42, 13.8)	1	29.60	5	10.88 (2.84, 16.72)
Respiratory System	7	10.97 (0.16, 18.18)	2	9.97 (0.02, 19.91)	9	10.97 (0.16, 18.18)
Sensory Organs	2	17.13 (14.61, 19.65)	3	10.99 (0.00, 16.53)	5	14.61 (10.99, 16.53)
Total	41	7.73 (1.07, 11.36)**	32	11.48 (7.23, 20.71)**	73	9.70 (2.75, 16.24)

Notes: IQR = Interquartile Range

§§Statistically significant difference between therapeutic classes: Alimentary Tract and Metabolism and Cardiovascular System (p = 0.005)

** Statistically significant difference between FDC and single drug NME same and different applicant (p = 0.005)

## Discussion

This study analyzed trends in all FDA approved FDCs in the period 1980–2012 and assessed the extent to which FDC drugs expand the effective patent and exclusivity life of previously marketed single active ingredient drugs. Study findings reveal that approval of FDC increased significantly over the last twenty years and varied by therapeutic class; the largest number of FDC approvals were for the treatment of highly prevalent conditions (i.e. cardiovascular and respiratory system drugs).

The USPTO may grant a patent claiming a FDC, whoever, the combination *per se* does not necessarily precludes a positive decision by the USPTO; the FDC must be innovative, showing evidence of unpredictability in combining different drugs and unexpected outcomes resulting from the use of the combination; non-obvious or different than previous art combinations of the drugs contained in the FDA; and useful or producing meaningful health effects.

Antiinfective FDC drugs entered the market relatively soon after the approval of the single active ingredient drug NMEs. This strategy may be related with the significantly longer effective patent and exclusivity life of antiinfectives, compared to other therapeutic classes, and the high demand of antiinfective drugs [23,24]. Additionally, most antiinfective FDC drugs are HIV antiretrovirals; these drugs are used in combination. If a company delays the patent application for a FDC, it is likely that clinicians will start using the combination of single active ingredients immediately after the single ingredients enter the market. The USPTO will not consider as non-obvious a FDC that has been used in clinical practice for several years before submission of the patent.

FDC drugs for cardiovascular diseases also entered the market relatively soon after the approval of the single active ingredient drug NMEs, and represent a significantly shorter increase in the effective patent and exclusivity life.

Study findings also evidence that pharmaceutical companies market FDC drugs shortly before the generic version of the single active ingredient drug enters the US market thus, extending the patent and marketing exclusivity protection of the single drugs included in the combination. In addition, approximately 80% of non-NME-FDC drugs were sponsored by the same applicant of at least one single ingredient drug included in the combination

The time lag between approval of the single active ingredient drug NMEs and the FDC and the increase in the effective patent and exclusivity life of non-NME-FDC drugs differed significantly between those non-NME-FDC sponsored by the same company and those sponsored by different companies. When sponsored by different companies, FDC drugs cannot enter the market before the expiration of the patents and exclusivities of the single active ingredients. Whereas, when sponsored by the same company, the pharmaceutical company can market their FDC drugs prior to generic entry, expanding the patent and market exclusivity protection of the active ingredients included in the combination.

FDC drugs simplify drug regimens, are more convenient for patients and may reduce out-of-pocket cost, and potentially increase adherence to treatments [25–28]. FDC drugs are generally recommended when the cost is assumable by third party payers and patients [29]. FDC therapies have become the standard of care for several diseases including HIV, type 2 diabetes, and several cardiovascular diseases. However, concerns have been raised regarding the effect of secondary patents on drug cost and affordability [15, 30–32]

FDC drugs allow pharmaceutical companies to maintain market share as the single active ingredient drugs lose patent protection and generic drugs enter the market increasing competition, and driving prices down [27,33,34]. Shifting the demand to FDC drugs as patents and exclusivities of single active ingredients expire may impose a financial burden on public and private health programs and patients [27,35–37]. Research is needed to assess the cost-effectiveness of FDC compared to single active ingredient pharmaceuticals.

## Limitations

Study results must be considered with a few caveats in mind. The study analyzed NMEs and BLAs; other biologic products including blood, vaccines, allergenics, tissues, and cellular and gene therapies were excluded from the analysis. The study includes FDC with at least one NME approved in the US in the period 1980–2012. The study includes the last patent listed in the FDA’s Orange Book for the first product number of the first NDA of each NME and excludes successive NDAs (e.g. line extensions). Study data used in the analysis are right censored. The effective patent and exclusivity life of NMEs can increase due to new patents listed by the sponsor’s company, patent extensions and pediatric exclusivity.

This study focuses on FDCs and active ingredients contained in the FDC. Future research should compare patents and marketed exclusivities, and prices differences between FDC drugs and single active pharmaceutical ingredients.

## Conclusion

Approvals of FDC drugs significantly increased over the last twenty years and varied by therapeutic class. The large majority of FDC includes at least one single active ingredient first approved by the FDA in the period of 1980–2012. A small percentage of FDC was approved using the FDA priority review procedure.

The time lag between first approval of the single active ingredients and FDC drug approval significantly varied by therapeutic class and sponsor’s company of the pharmaceutical product. Likewise, the time lag in the market entry between the FDC and single generic drugs vary significantly depending on whether the sponsor of the FDC and the single active ingredients included in the combination are the same or different. Pharmaceutical companies market FDC drugs shortly before the generic alternative of the single active ingredient in the combination reaches the market, thus effectively extending the patent and marketing exclusivity life of the single drugs included in the combination. The difference in the effective patent and exclusivity life between FDC and single ingredient drugs vary significantly depending on the therapeutic class and whether the sponsor of the FDC and the single active ingredients included in the combination were the same or different.
